# Supplementary material for: Clinical and Humanistic Outcomes of Community Pharmacy-Based Healthcare Interventions Regarding Medication Use in Older Adults: A Systematic Review and Meta-Analysis
Source: Healthcare (Basel). 2021 Nov 18;9(11):1577. doi: 10.3390/healthcare9111577 (PMC8625440; doi:10.3390/healthcare9111577)
Supplement: Supplementary file 1 [file healthcare-09-01577-s001.zip › healthcare-1406608-supplementary.pdf]

Table S1. Search Strategy.

Databases	Search strategy
Ovid Medline/Ovid Embase/APA PSY Info	1. exp Community Pharmacy Services/ or “community pharmac*” .mp.<br><br>2. “retail pharmac* “.mp. [mp=title, abstract, original title, name of substance word, subject heading word, floating sub-heading word, keyword heading word, organism supplementary concept word, protocol supplementary concept word, rare disease supplementary concept word, unique identifier, synonyms]<br>3. “independent pharmac*” .mp.<br>4. exp Pharmacists/ or "outpatient pharmac*".mp.<br>5. exp Pharmacists/<br>6. (pharmacy or pharmacies*).mp. [mp=title, abstract, original title, name of substance word, subject heading word, floating sub-heading word, keyword heading word, organism supplementary concept word, protocol supplementary concept word, rare disease supplementary concept word, unique identifier, synonyms]<br>7. 1 or 2 or 3 or 4 or 5 or 6<br>8. exp “Delivery of Health Care”/ or strateg*.mp.<br>9. initiat*.mp.<br>10. exp Pharmacies/<br>11. service*.mp.<br>12. service based.mp. or exp Primary Health Care/<br>13. exp “Activities of Daily Living”/ or activit*.mp.<br>14. exp Patient Reported Outcome Measures/ or exp Treatment Outcome/ or exp Outcome Assessment, Health Care/ or exp “Outcome and Process Assessment, Health Care”/ or outcome*.mp. or exp Patient Outcome Assessment/<br>15. patient compliance.mp. or exp Patient Compliance/<br>16. medication adherence.mp. or exp Patient Compliance/ or exp Medication Adherence/<br>17. concordance.mp.<br>18. exp Patient Compliance/ or patient* adherence.mp. or exp Medication Adherence/<br>19. medic* outcome.mp.<br>20. 14 or 15 or 16 or 17 or 18 or 19<br>21. geriatric*.mp.<br>22. exp Middle Aged/ or elder*.mp. or exp Aging/ or exp Aged/ or exp "Aged, 80 and over"/<br>23. exp Aged/ or old* adult*.mp.<br>24. exp Aged/ or exp “Aged, 80 and over”/ or aged 65 year*.mp. or exp Middle Aged/<br>25. community dwell*.mp.<br>26. 21 or 22 or 23 or 24 or 25<br>27. 8 or 9 or 10 or 11 or 12 or 13<br>28. 7 and 20 and 26 and 27<br>29. limit 28 to ((“all aged (65 and over)” or “aged (80 and over)”) and english and last 10 years)
Scopus	TITLE-ABS-KEY("community pharmac*" OR "retail pharmac*" OR "independent pharmac*" OR "outpatient pharmac*") AND (intervention* OR

	strateg* OR initiati* OR activit* OR program* OR service* OR "service-based") AND (outcome* OR "outcome assessment*" OR "treatment outcome*" OR "medication adherence" OR "patient compliance" OR "patient adherence" OR "medication compliance" OR "medication outcome" OR concordance) AND (geriatric* OR old* OR "old* adult*" OR aging OR elder* OR aged OR "aged 65 year*" OR "community dwell*") AND (LIMIT-TO (PUBYEAR,2021) OR LIMIT-TO (PUBYEAR,2020) OR LIMIT-TO (PUBYEAR,2019) OR LIMIT-TO (PUBYEAR,2018) OR LIMIT-TO (PUBYEAR,2017) OR LIMIT-TO (PUBYEAR,2016) OR LIMIT-TO (PUBYEAR,2015) OR LIMIT-TO (PUBYEAR,2014) OR LIMIT-TO (PUBYEAR,2013) OR LIMIT-TO (PUBYEAR,2012) OR LIMIT-TO (PUBYEAR,2011) OR LIMIT-TO (PUBYEAR,2010)) AND (LIMIT-TO (SUBJAREA,"MEDI") OR LIMIT-TO (SUBJAREA,"PHAR") OR LIMIT-TO (SUBJAREA,"NURS") OR LIMIT-TO (SUBJAREA,"SOCI")) AND (LIMIT-TO (LANGUAGE,"English")) AND (LIMIT-TO (freetoread,"all")) AND (LIMIT-TO (SRCTYPE , "j"))
CINAHL	S11 S1 AND S2 AND S5 AND S6Limiters - Publication Year: 2010-2020<br>Narrow by Language: - english<br>Narrow by SubjectAge: - aged, 80 & over<br>Narrow by SubjectAge: - aged: 65+ years<br>S10 S1 AND S2 AND S5 AND S6Narrow by Language: - english<br>Narrow by SubjectAge: - aged, 80 & over<br>Narrow by SubjectAge: - aged: 65+ years<br>S9 S1 AND S2 AND S5 AND S6Narrow by SubjectAge: - aged, 80 & over<br>Narrow by SubjectAge: - aged: 65+ years<br>S8 S1 AND S2 AND S5 AND S6Narrow by SubjectAge: - aged: 65+ years<br>S7 S1 AND S2 AND S5 AND S6<br>S6 S3 OR S4<br>S5(elderly or aged or older or elder or geriatric or elderly people or old people or old people or senior) OR (older adults or elderly or geriatric or geriatrics or aging or senior or seniors or older people or aged 65 or 65+)<br><br>S4 MH (health outcomes or health impacts or health effects or effects on health) OR ("health impact*" OR "medication outcome*" OR "outcome assessment*" OR "treatment outcome*")<br>S3 MH (patient compliance or patient adherence) OR MH (medication adherence or medication compliance) OR (medic* compliance OR medic* adherence OR concordance OR patient adherence OR patient persistence)<br>S2 service* OR program* OR initiative* OR intervention* OR "service-based" OR activit*<br>S1 MH community pharmacy services OR ("community pharmac*" OR 'outpatient pharmac*" OR "independent pharmac*" OR "retail pharmac*
